# Obesity-induced neuroinflammation and cognitive impairment in young adult versus middle-aged mice

**DOI:** 10.1186/s12979-022-00323-7

**Published:** 2022-12-22

**Authors:** Rosemary E. Henn, Sarah E. Elzinga, Emily Glass, Rachel Parent, Kai Guo, Adam M. Allouch, Faye E. Mendelson, John Hayes, Ian Webber-Davis, Geoffery G. Murphy, Junguk Hur, Eva L. Feldman

**Affiliations:** 1 Department of Neurology, University of Michigan, Ann Arbor, MI 48109 USA; 2 NeuroNetwork for Emerging Therapies, University of Michigan, Ann Arbor, MI 48109 USA; 3 Michigan Neuroscience Institute, University of Michigan, Ann Arbor, MI 48109 USA; 4 Department of Molecular and Integrative Physiology, Division of Cardiovascular Medicine, University of Michigan, Ann Arbor, MI 48109 USA; 5 Department of Biomedical Sciences, University of North Dakota, Grand Forks, ND 58202 USA

**Keywords:** Aging, Cognitive impairment, High-fat diet, Obesity, Mouse, Inflammation, Brain, RNA-seq

## Abstract

**Background:**

Obesity rates are increasing worldwide. Obesity leads to many complications, including predisposing individuals to the development of cognitive impairment as they age. Immune dysregulation, including inflammaging (*e.g.*, increased circulating cytokines) and immunosenescence (declining immune system function), commonly occur in obesity and aging and may impact cognitive impairment. As such, immune system changes across the lifespan may impact the effects of obesity on neuroinflammation and associated cognitive impairment. However, the role of age in obesity-induced neuroinflammation and cognitive impairment is unclear. To further define this putative relationship, the current study examined metabolic and inflammatory profiles, along with cognitive changes using a high-fat diet (HFD) mouse model of obesity.

**Results:**

First, HFD promoted age-related changes in hippocampal gene expression. Given this early HFD-induced aging phenotype, we fed HFD to young adult and middle-aged mice to determine the effect of age on inflammatory responses, metabolic profile, and cognitive function. As anticipated, HFD caused a dysmetabolic phenotype in both age groups. However, older age exacerbated HFD cognitive and neuroinflammatory changes, with a bi-directional regulation of hippocampal inflammatory gene expression.

**Conclusions:**

Collectively, these data indicate that HFD promotes an early aging phenotype in the brain, which is suggestive of inflammaging and immunosenescence. Furthermore, age significantly compounded the impact of HFD on cognitive outcomes and on the regulation of neuroinflammatory programs in the brain.

**Supplementary Information:**

The online version contains supplementary material available at 10.1186/s12979-022-00323-7.

## Background

The obesity crisis is reaching pandemic levels [1]. According to the World Health Organization, in 2016 over 109 billion adults worldwide were overweight or obese. Not only does obesity significantly impact quality of life [2–4], but it also promotes a multitude of systemic complications. Obesity leads to comorbidities including type 2 diabetes, cardiovascular disease, cancer, stroke, and cognitive impairment [1, 5–7]. Further, obesity is a known risk factor for aging associated dementias, including Alzheimer’s disease and Alzheimer’s disease related dementias. Studies demonstrate that mid-life obesity in particular is a risk factor for developing dementia later in life [5, 8]. However, obesity can occur throughout the lifespan, and its effects on cognition during adolescence and throughout adulthood are unclear. The impact of age on obesity-induced cognitive impairment requires better clarification. Further, the mechanistic link between obesity and cognitive impairment remains poorly characterized.

Immune dysregulation is a hallmark of both obesity [9] and Alzheimer’s disease [10], and contributes to obesity induced cognitive impairment [11, 12]. Obesity is associated with systemic [13] and central nervous system (CNS) inflammation, including in the hippocampus [11, 12, 14], a brain region responsible for learning and memory tasks affected by Alzheimer’s disease [15]. While it is evident that immune responses contribute to obesity-induced cognitive impairment, differential effects of age on this interaction are unclear. The immune system is profoundly impacted by aging, and effects of aging on both innate and adaptive immune function have been extensively studied [16–18]. The aging immune system has reduced ability to effectively mount responses to challenges, a phenomenon termed ‘immunosenescence’ [18]. Yet, the innate arm of the immune system becomes aberrantly overactive, leading to chronic low level systemic inflammation, termed ‘inflammaging’ [16, 17, 19]. Due to these age-dependent changes in immune responses, age likely impacts the role of inflammation in obesity-induced cognitive impairment.

Adolescent and adult murine models of diet-induced obesity demonstrate cognitive impairment [12, 20, 21], including increased anxiety-like behavior [22, 23]. Equivalent studies in aged mice are lacking; some evidence suggests that obesity worsens age-related cognitive decline [24], while other studies show that consuming a high-fat diet (HFD) does not affect baseline aging deficits [23]. However, it is established that HFD promotes the aging process in the CNS. Not only does HFD accelerate Alzheimer’s disease pathology and associated cognitive impairment [25–27], but it also exacerbates neuroinflammation and microglial aging in the healthy brain [28, 29]. Given this effect of HFD on CNS age-related inflammation, alongside the established role of the immune system in obesity-induced cognitive impairment, this study investigated potential differential effects of age on hippocampal neuroinflammation and cognitive function in obesity.

Herein, we initially employed our established model of HFD-induced obesity throughout adolescence and into adulthood to determine the effect of obesity on hippocampal transcriptomics. We found that obesity during earlier periods of the lifespan induced an ‘early-aging’ hippocampal phenotype. Therefore, we then used this same model in young adult and middle-aged mice to determine the impact of HFD and age on obesity-induced neuroinflammation and cognitive impairment. We found that age exacerbated HFD effects on a limited number of metabolic parameters. However, age significantly impacted the effect of HFD on fear conditioning cognitive performance. Further, age differentially affected hippocampal inflammatory gene expression, indicating that age plays an important role in the regulation of inflammatory responses in obesity.

## Results

### Obesity promotes a premature aging transcriptomic signature in the hippocampus

**Fig. 1:**
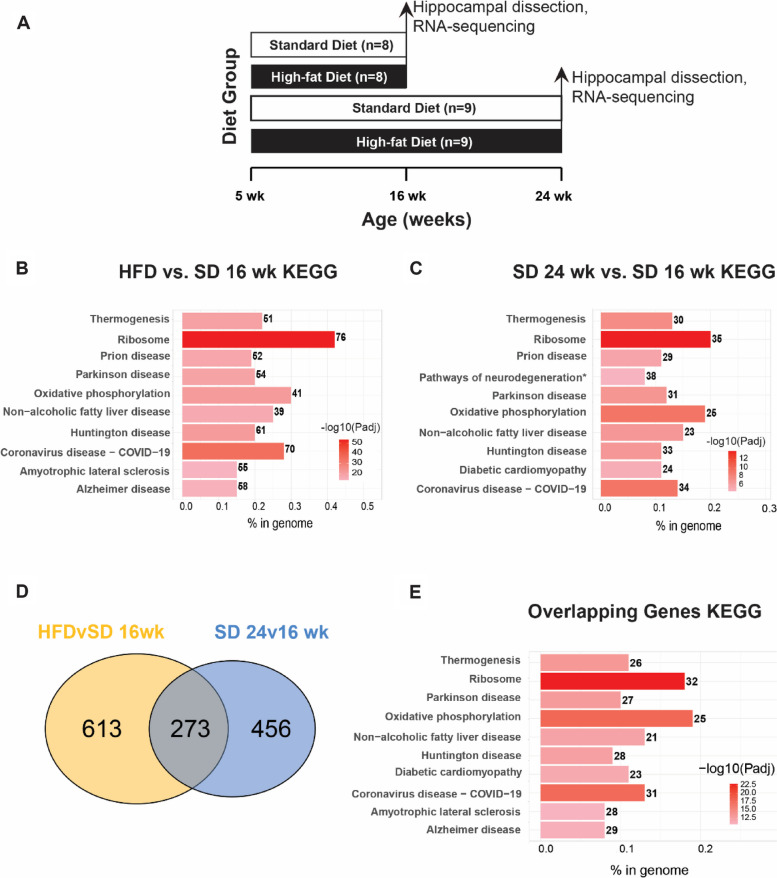
Obesity promotes a premature aging transcriptomic signature in the hippocampus. (**A**) Study paradigm for cohort 1: mice aged 5 wk were fed high-fat diet (HFD) or standard diet (SD) for 11 wk (final age 16 wk) or 19 wk (final age 24 wk) and hippocampi were analyzed by RNA-sequencing. (**B**) Bar plot of KEGG enrichment analysis of differentially expressed genes (DEGs) in HFD 16 wk versus SD 16 wk. (**C**) Bar plot of KEGG enrichment analysis of DEGs in SD 24 wk versus SD 16 wk DEGs. (**D**) Venn diagram of overlapping DEGs (adjusted *P*-value < 0.05; grey) in HFD 16 wk versus SD 16 wk comparison (yellow) with SD 24 wk versus SD 16 wk comparison (light blue). (**E**) Bar plot of KEGG enrichment analysis of the 273 overlapping DEGs from (D). For (B), (C), and (E), bar color represents -log_10_(Padj), number to the right of the bar represents number of DEGs in the KEGG pathway, and bar length along the x-axis, ‘% in genome,’ represents the fraction of DEGs relative to all KEGG pathway genes

First, our established mouse model of diet-induced obesity and cognitive decline [20, 30, 31] was used to determine the effect of chronic obesity on the hippocampal transcriptome in adolescent mice maturing into young adulthood. Hippocampi from a previously published study [31] using C57BL/6 mice (*n* = 8–9 per group) fed HFD (high fat diet) or SD (standard diet) from 5 weeks (wk) of age until either 16 or 24 wk of age (Fig. 1A; cohort 1) were processed for RNA-sequencing (RNA-seq). Hippocampal gene expression analysis identified 886 differentially expressed genes (DEGs; adjusted *P*-value < 0.05) between HFD and SD at 16 wk age, and 111 genes between HFD and SD at 24 wk (Additional Table 1). Interestingly, HFD mice had similar gene expression at both ages, indicating that HFD-related changes likely occur early and are persistent. Next, Kyoto Encyclopedia of Genes and Genomes (KEGG) pathway enrichment analysis was performed to infer potential biological significance of the DEGs. The identified DEGs between HFD and SD in 16 wk young adult mice were enriched in pathways related to metabolism (*e.g.,* ‘oxidative phosphorylation,’ ‘non-alcoholic fatty liver disease’) and neurodegenerative disease (*e.g.,* ‘Parkinson disease,’ ‘Alzheimer disease’) (Fig. 1B, top 10 pathways; Additional Table 1).

There was also an effect of age alone on the hippocampal transcriptome. In SD mice, age (24 wk versus 16 wk) affected 729 genes. KEGG pathway analysis of age-related DEGs identified enrichment of pathways related to metabolic dysfunction (*e.g.,* ‘oxidative phosphorylation,’ and ‘diabetic cardiomyopathy’) and neurodegenerative disease (*e.g.,* ‘Parkinson disease’ and ‘Huntington disease’) (Fig. 1C, top 10 enriched pathways; Additional Table 1). Because the KEGG pathways identified for HFD versus SD DEGs at 16 wk mirrored those for the age associated DEG set in young versus mature adult SD mice, the impact of HFD on age related gene expression was assessed. Indeed, 273 genes overlapped between the 16 wk HFD versus SD DEGs and the SD 24 versus 16 wk DEGs (Fig. 1D). KEGG pathway analysis for these 273 genes revealed enrichment similar to the diet- and age-dependent DEGs, including ‘ribosome,’ ‘oxidative phosphorylation,’ and ‘Parkinson disease’ (Fig. 1E, top ten enriched pathways; Additional Table 1).

### HFD induces obesity and metabolic dysfunction in adult and aged mice

**Fig. 2:**
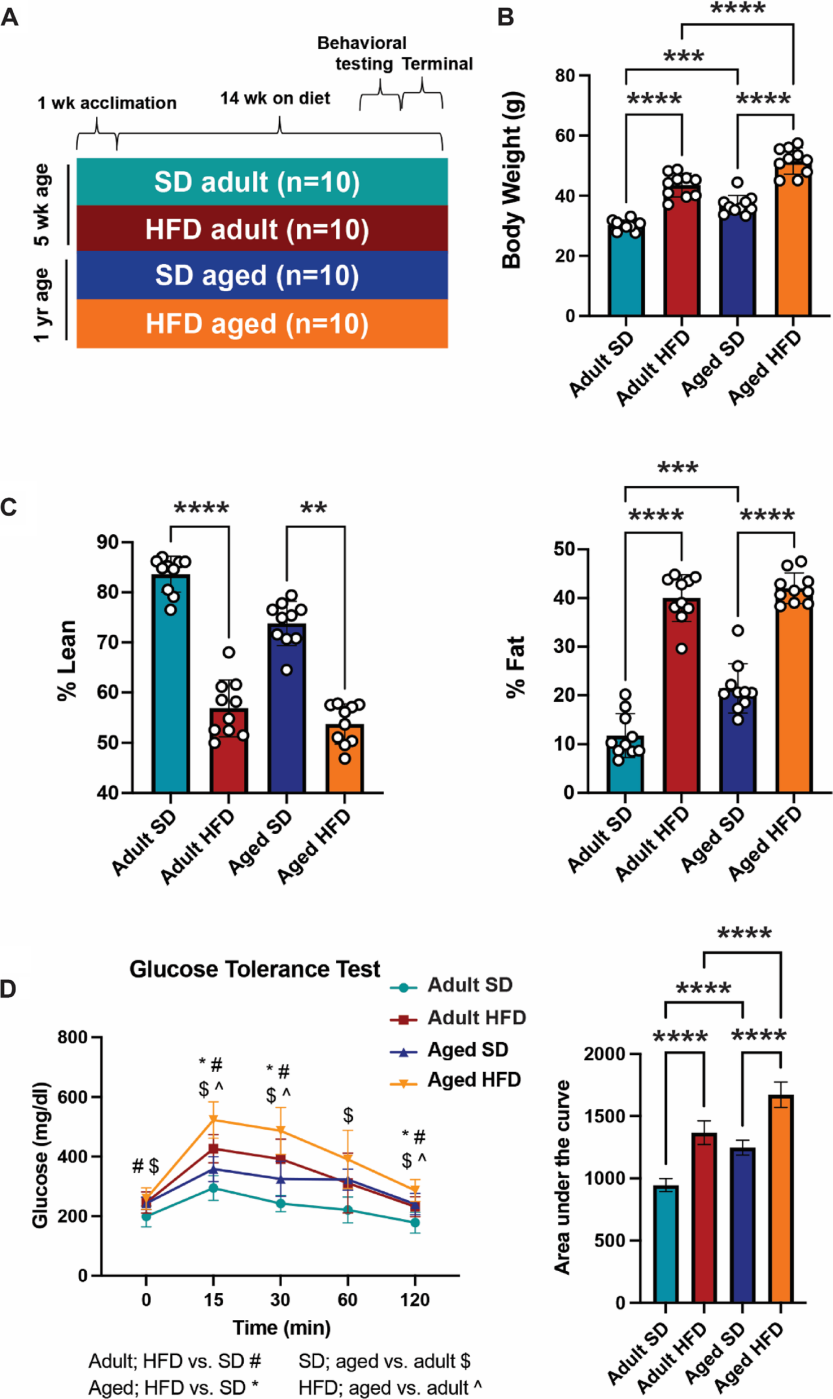
HFD induces obesity and metabolic dysfunction in adult and aged mice. (**A**) Study design for cohort 2: mice aged 5 wk and 1 yr were fed high-fat diet (HFD) or standard diet (SD) for 14 wk; SD adult in teal, HFD adult in red, SD aged in blue, and HFD aged in orange. (**B**) Terminal body weights at study endpoint, ***P < 0.001, ****P < 0.0001 by one-way ANOVA. (**C**) Terminal body composition by percent lean and fat mass, **P < 0.01, ***P < 0.001, ****P < 0.0001 by Kruskal–Wallis for non-normally distributed lean mass and by one-way ANOVA for fat mass. (**D**) Terminal glucose tolerance test, *P* < 0.05 by two-way ANOVA (*, #, $, ^; shown in legend); area under the curve for each experimental group, ****P < 0.0001 by one-way ANOVA. *n* = 10 per group for all measures. Data are presented as mean ± standard deviation

Since obesity induced this premature aging hippocampal phenotype, we next investigated diet-induced obesity in young adult (termed ‘adult’) and middle-aged (termed ‘aged) mice to determine the effect of age on obesity-induced neuroinflammation and cognitive impairment. Adult and aged mice were fed HFD or SD for 14 wk (Cohort 2; Fig. 2A). HFD mice had significantly higher terminal body weights than their SD counterparts, regardless of age (adult HFD versus adult SD and aged HFD versus aged SD; *P* < 0.0001, one-way ANOVA) (Fig. 2B). Further, older age was associated with increased body weight, regardless of diet (adult SD versus aged SD, *P* < 0.001; adult HFD versus aged HFD, *P* < 0.0001). Next, we measured terminal body composition, as percent lean and percent fat mass. Relative to their SD counterparts, both adult and aged HFD mice had lower percentage of lean body mass (adult HFD versus adult SD, *P* < 0.0001; aged HFD versus aged SD, *P* = 0.006, one-way ANOVA) and a higher percentage of fat mass (adult HFD versus adult SD, *P* < 0.0001; aged HFD versus aged SD, *P* < 0.0001) (Fig. 2C). Further, aged SD mice had a higher percentage body fat than adult SD mice (*P* = 0.0001), but there was no difference in percent fat between the adult and aged HFD groups. Terminal glucose tolerance tests were then performed to determine the effect of HFD on glucose homeostasis in adult and aged mice (Fig. 2D). Both adult and aged HFD mice showed an impaired response to glucose challenge compared to SD counterparts, demonstrated by higher peak blood glucose levels at 15 min post glucose bolus and increased area under the curve (AUC). Older age was associated with a worse response to glucose challenge in both SD and HFD animals (AUC, adult SD versus aged SD, *P* < 0.0001; adult HFD versus aged HFD, *P* < 0.0001, one-way ANOVA).

Next, to further define the differential effect of obesity on the metabolic health of adult versus aged mice, we quantified plasma fasting insulin levels, as well as terminal epididymal adipocyte hypertrophy (Additional Fig. 1) and hepatic pathology (Additional Fig. 2) to assess the effect in multiple organs. HFD reduced the proportion of small adipocytes in both adult and aged mice (Additional Fig. 1A). Age also affected the distribution of adipocyte size in controls, with a lower proportion of small adipocytes in aged SD mice versus adult SD animals. However, older age did not compound the effects of HFD on adipocyte hypertrophy, suggesting a potential obesity ‘ceiling effect’ in response to HFD. HFD elevated plasma insulin concentrations in adults (*P* = 0.0007, Kruskal–Wallis), but in HFD aged mice the increase relative to SD mice did not reach statistical significance (*P* = 0.296) (Additional Fig. 1B). Kleiner scoring (32–34) for non-alcoholic fatty liver disease (NAFLD) pathology demonstrated that HFD increased steatosis in both adult (*P* = 0.0004, Kruskal–Wallis) and aged (*P* = 0.039) mice, but corresponding increases in lobular inflammation did not reach statistical significance (Additional Fig. 2B,C). Further, the NAFLD activity score, a summation of steatosis, lobular inflammation, and ballooning degeneration, was higher in HFD relative to SD in both adult (*P* = 0.0051, Kruskal–Wallis) and aged (*P* = 0.0197) mice (Additional Fig. 2D). In addition to Kleiner scoring, macrosteatosis was quantified by droplet counts, demonstrating that HFD increased counts in both adult (*P* = 0.0021, Kruskal–Wallis) and aged (*P* = 0.042) mice (Additional Fig. 2E).

### HFD increases plasma but not hippocampal inflammatory cytokines

**Fig. 3:**
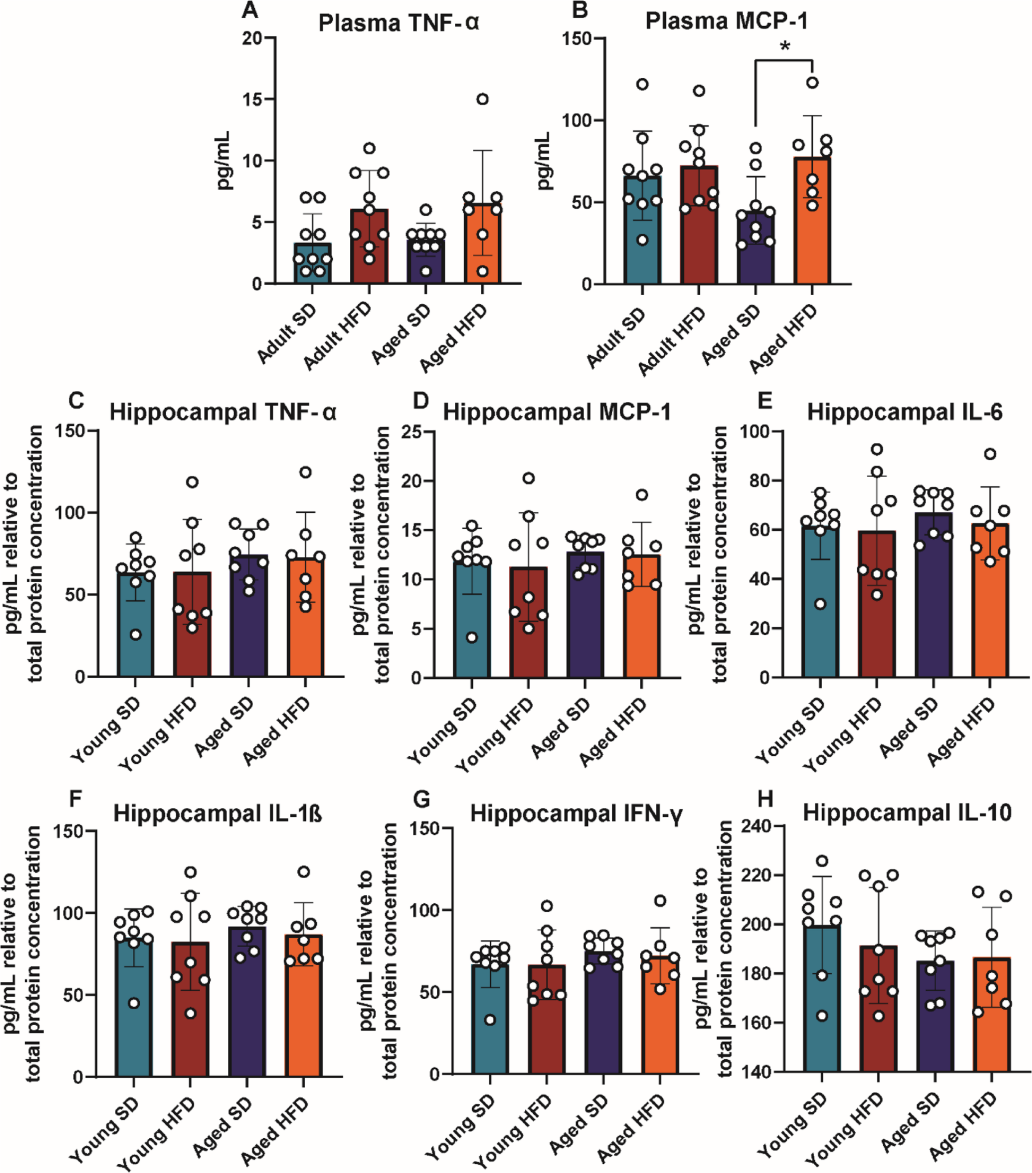
Terminal plasma and hippocampal cytokines. Cohort 2 plasma and hippocampal cytokine protein concentrations via enzyme-linked immunosorbent assay (ELISA) for plasma (**A**) TNF-α and (**B**) MCP-1, and hippocampal (**C**) TNF-α, (**D**) MCP-1, (**E**) IL-6, (**F**) IL-1β, (**G**) IFN-γ, and (**H**) IL-10; *n *= 7–9 per group. For all bar plots, adult SD (teal), adult HFD (red), aged SD (blue), aged HFD (orange); *P < 0.05 by one-way ANOVA; error bars represent mean ± standard deviation

To examine possible differences in inflammatory cytokine and chemokine production due to HFD in adult and aged mice, plasma and hippocampi were assessed via ELISA (Fig. 3; cohort 2). In plasma, there were elevated concentrations of the pro-inflammatory chemokine monocyte chemoattractant protein-1 (MCP-1) in aged HFD mice relative to aged SD animals (Fig. 3B; *P* = 0.0235, one-way ANOVA). Adult HFD and adult SD mice had similar plasma MCP-1 levels. Tumor necrosis factor alpha (TNF-α) plasma concentrations tended to be higher in HFD mice of both ages, although this did not reach statistical significance (Fig. 3A). There were no differences in any of the measured hippocampal cytokines across the groups (Fig. 3C-H).

### HFD increases hippocampal microglial numbers

To better understand pro-inflammatory changes due to HFD and age, we next measured changes in microglial numbers in the hilus (Cohort 3; Additional Fig. 3). We observed that adult HFD mice had increased microglial numbers in this region of the hippocampus compared to adult SD mice (Additional Fig. 3A). Age alone appeared to also increase microglial numbers; however this was non-significant. There was also a nominal increase in microglial numbers in aged HFD vs aged SD animals. Interestingly, this increase in microglial numbers had a moderate positive correlation with body weight, where increasing body weights were associated with increased hilus microglial numbers (Additional Fig. 3B).

### HFD alters fear responses, particularly in aged mice

**Fig. 4:**
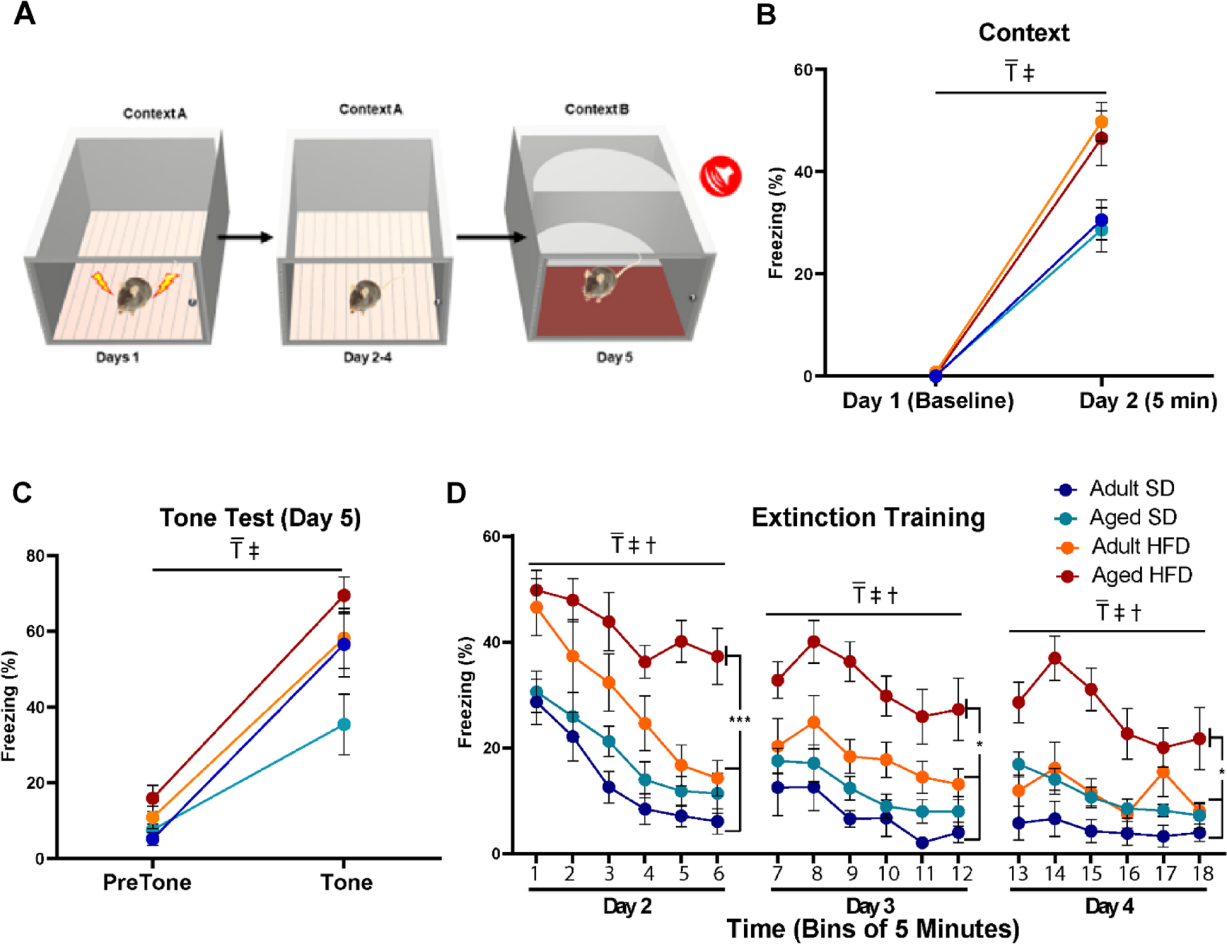
HFD increases fear responses, particularly in aged mice. Aged HFD mice exhibit deficits in extinction learning (*n* = 10/group). (**A**) Fear conditioning paradigm. (**B**) Context test. Compared to baseline all mice displayed significant levels of freezing when returned to the training context on day 2 (3-way RM ANOVA; main effect of training F (1, 36) = 315.7; ₸, *p* < 0.0001). Freezing levels appeared to be modulated by diet (3-way RM ANOVA; main effect of diet F (1, 36) = 18.8; ‡, *p* < 0.0001) which was driven by the segregation of the two diet groups regardless of age at 24 h (1-way ANOVA F (3, 36) = 6.096, *p* = 0.0018). (**C**) Tone test. Mice were placed in a novel context and after a 180 s baseline period were exposed to the same tone used during training (the last 30 s of baseline and first 30 s of tone are presented. A 3-way repeated measures ANOVA revealed a main effect of training (F (1, 36) = 147.5 ₸, *p* < 0.0001). Freezing levels appeared to be modulated by diet (3-way RM ANOVA; main effect of diet F (1, 36) = 18.8; ‡, *p* < 0.0054) but this effect was not specific to group, although when freezing in response to tone was analyzed, there was a significant difference between mice in the Aged SD group and the Aged HFD group (1-way ANOVA F (3, 36) = 3.593,*P* = 0.0228 followed by Tukey post hoc comparison: adjusted *p* = 0.0141). (**D**) Extinction training. On days 2, 3 and 4 mice were returned to the original training context and freezing was measured for 30 min (presented here in 5 min bins). Across all three days there was a reduction in freezing in response to repeated context exposure (3-way RM ANOVA main effect of training: Day 2 F (1, 36) = 157.2, ₸, *p* < 0.0001; Day 3: F (1, 36) = 14.17; ₸, *p* = 0.0006; Day 4 F (1, 36) = 11.84; ₸, *p* = 0.0015) which was likely influenced by diet (3-way RM ANOVA main effect of diet: Day 2 F (1, 36) = 22.46, ₸, *p* < 0.0001; Day 3: F (1, 36) = 14.02; ₸, *p* = 0.0006; Day 4 F (1, 36) = 10.63; ₸, *p* = 0.0024) and age (3-way RM ANOVA main effect of age: Day 2 F (1, 36) = 4.963, ₸, p = 0.0322; Day 3: F (1, 36) = 6.750; ₸, *p* = 0.0135; Day 4 F (1, 36) = 16.03; ₸, *p* = 0.0003). This was especially evident on Day 2 where a training x diet x age interaction was observed (F (1, 36) = 4.963*p* = 0.0322). To more directly examine the effectiveness of extinction training a 1-way ANOVA was used to analyze freezing levels recorded in the final bin on each day. Across all three days there was a main effect of group (Day 2: F (3, 36) = 12.69, *p* < 0.0001; Day 3: F (3, 36) = 7.524, *p* = 0.0005; Day 4: F (3, 36) = 5.530 *P* = 0.0032). This effect appears to be a function of mice in the Aged HFD group which exhibited significantly more freezing as compared to the other 3 groups (Tukey’s multiple comparisons test *** *p* < 0.001, * *p* < 0.05). All data are presented as mean ± SEM

To assess the impact of HFD and aging on cognition, associative learning was evaluated using a Pavlovian fear conditioning paradigm (Fig. 4; cohort 2). After a baseline period and three tone-shock pairings in the conditioning chambers on day 1, mice were returned to the same chambers on day 2. Freezing was then measured as an index of associative memory between the chamber and the aversive foot shock. During the first 5 min in the training context on day 2, all mice exhibited robust freezing (Fig. 4B; *P* < 0.0001, 3-way RM ANOVA). However, HFD mice froze more than SD mice, regardless of age (*P* = 0.0001, 3-way RM ANOVA). When placed in a novel context and exposed to the same tone used during training but in the absence of a foot-shock on day 5, all mice froze significantly more when compared to freezing during the pre-tone baseline period (Fig. 4C; *P* < 0.0001, 3-way RM ANOVA). Additionally, a main effect of diet was observed, where HFD mice froze more compared to SD mice (*P* = 0.0054, 3-way RM ANOVA). When analyzing tone data, there was a compounding effect of age on diet, where aged HFD mice froze more compared to aged SD mice (*P* = 0.0141, 1-way ANOVA). On days 2 through 4 when mice were returned to the original training context for 30 min/day (Fig. 4D), HFD mice initially displayed higher levels of freezing (Bin 1 in Fig. 4D is the Day 2 data presented in Fig. 4B). During the 30 min of extinction training on day 2, a main effect of extinction training was observed (*P* < 0.0001, 3-way RM ANOVA). There were also main effects of both diet and age on extinction freezing, where HFD mice froze more compared to SD mice and aged mice froze more compared to adults (Fig. 4D; P values dependent upon day and Bin and are detailed in figure legend). Furthermore, aged HFD mice froze more than all other groups, particularly for the final bin of each day (Day 2, *P* < 0.0001; Day 3, *P* = 0.0005; Day 4, *P *= 0.0032; 1-way ANOVA).

### Age determines hippocampal transcriptomic inflammatory response to HFD

**Fig. 5:**
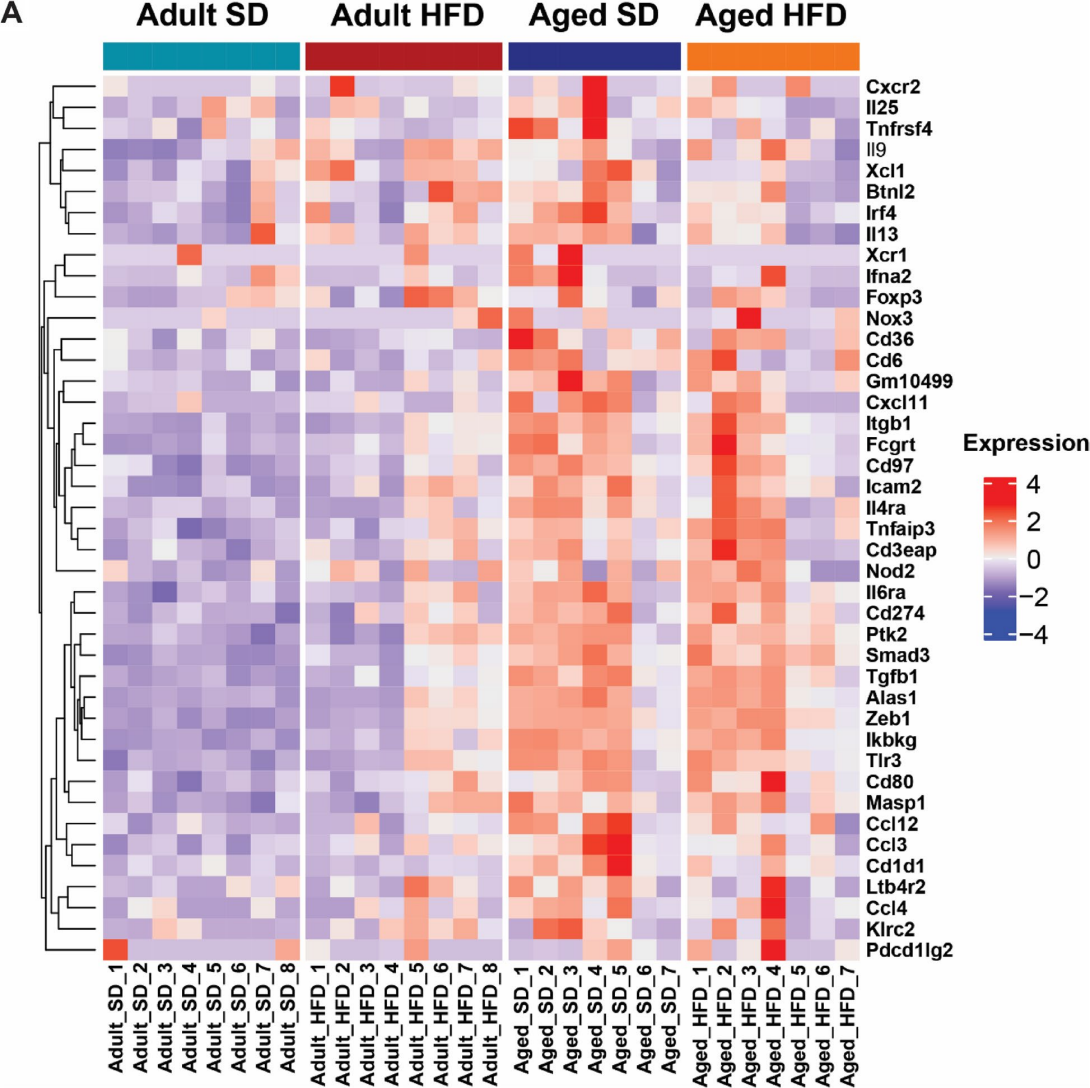
Age determines hippocampal transcriptomic response to obesity. Heat map of hippocampal gene expression counts measured by NanoString nCounter for differentially expressed genes (diet*age interaction by mixed-effects model, *P*-value < 0.05); adult standard diet (SD; teal, first column), adult high-fat diet (HFD; red, second column), aged SD (blue, third column), and aged HFD (orange, fourth column). Genes (rows) were hierarchically clustered, while columns include animals ordered by replicate number (*n* = 7–8 per group). Color represents relative expression levels of normalized counts for each gene across all samples from low expression (blue) to high expression (red)

Hippocampal inflammatory gene expression profiling by NanoString nCounter assay revealed a differential effect of diet dependent upon age (Fig. 5; cohort 2). A pattern of relative gene expression emerged for many of the DEGs due to age and diet (*n* = 32 DEGs, *P* < 0.05), with increased expression in HFD adults versus SD adults, but decreased expression in aged HFD mice relative to age-matched SD controls. Specifically, HFD in adult animals frequently increased expression of inflammatory genes with a significant increase in 18% of DEGs. Age increased gene expression even further, with aged SD animals exhibiting significantly higher expression in 60% of inflammatory DEGs. However, HFD in aged animals decreased the mean expression of many of these same genes, with significant decrease in 60% of DEGs. These results indicate bi-directional effects from HFD; HFD increased expression of inflammatory genes in adults but decreased expression in aged mice relative to age-matched SD controls. Of these genes, many were related to either lymphocyte differentiation or function (Additional Table 2). Additionally, a large proportion were also involved in chemotaxis or inflammation, and innate immune cell activation or pattern recognition. Genes of interest within these broad functions included C-X-C motif chemokine 11 (*CxCl11*), zinc finger E-box-binding homeobox 1 (*Zeb1*), and interferon regulatory factor-4 (*Irf4*). Altogether, these data indicate that HFD impacts expression of genes involved in immune cell recruitment, activation, and function, in an age-dependent manner.

## Discussion

Immune dysregulation is a common feature in obesity and aging, which is thought to contribute to pathological changes in the CNS, including cognitive impairment. Here, in a mouse model of obesity and cognitive impairment [20, 30], HFD promoted a premature aging signature in the young adult hippocampus. This premature aging signature prompted us to examine differential effects of HFD with age. Age exacerbated effects of HFD on cognition, as measured by fear conditioning. Age also worsened HFD effects on body weight and glucose tolerance. However, most metabolic phenotyping parameters (body composition, plasma insulin, adipocyte hypertrophy, NAFLD liver pathology) appeared to reach a ‘ceiling’ where HFD produced similar effects in both adult and aged mice. Additionally, HFD induced bi-directional hippocampal inflammatory gene expression changes: increased expression in adults but decreased expression in aged animals. Genes with this bi-directional regulation were broadly related to lymphocyte differentiation or function, chemotaxis or inflammation, and innate immune cell activation or pattern recognition. Overall, our data indicate that age plays an important role in obesity-induced hippocampal inflammatory response and cognitive impairment.

Here, HFD promoted a premature hippocampal aging phenotype. Many DEGs in the hippocampus of 16 wk old HFD versus SD 16 mice were also DEGs arising from aging in controls (SD mice, 24 wk vs 16 wk of age). These overlapping genes were highly enriched in pathways related to metabolism, ribosome, oxidative phosphorylation, and neurodegenerative diseases, including Alzheimer’s Disease. In the brain, in addition to neurodegenerative disease and metabolic pathways, ribosome and oxidative phosphorylation pathways are considered to be aging signatures in multiple cell types [32]. Our findings align with existing evidence demonstrating that many of the consequences of aging, *i.e.* inflammaging, cellular senescence, telomere shortening, and genomic damage are also implicated in obesity and metabolic dysfunction [19, 33]. Others have similarly shown obesity induces an aging phenotype [34], particularly in adipose tissue [35]. This perhaps unsurprising as immune function and metabolism, both classically dysregulated during aging, are intrinsically linked. For example, saturated fatty acids activate pattern recognition receptors, such as toll-like receptors [36, 37], creating a pro-inflammatory state. In turn, this pro-inflammatory state directly impacts insulin signaling, promoting insulin resistance [37, 38].

Regardless of age, HFD mice had greater fat mass and lower lean mass versus SD controls. HFD mice also had fewer small adipocytes, increased liver steatosis, macrosteatosis, and NAFLD score. Although not significant, aged HFD mice also had higher plasma insulin concentrations. These results are expected, since we [20, 30, 31] and others [39, 40] have shown HFD consistently causes obesity, dysregulated glucose metabolism, liver pathology, and adipocyte hypertrophy. Interestingly, for weight and glucose tolerance, age compounded dietary effects; HFD and age worsened the metabolic phenotype relative to adult controls, where HFD aged mice displayed the most severe phenotype. However, for all other parameters, aged and adult HFD mice had a similar metabolic phenotype, possibly due to a ‘ceiling effect’ reaching a plateau with HFD, which older age could not worsen past a certain point.

Maintaining proper metabolic support in the CNS, including via neuron/glia metabolic crosstalk, is critical for normal cognitive function [41, 42]. We and others have shown that obesity and metabolic dysfunction cause CNS metabolic changes, including CNS insulin resistance [20, 43] and hypoglycemia [44, 45]. Furthermore, systemic metabolic changes can indirectly impact the CNS via blood brain barrier disruption [46, 47]. This disruption allows for increased entry of peripheral factors, such as inflammatory mediators, into the brain [46] and further contributes to neurodegeneration and cognitive impairment. Indeed, chronic HFD impairs cognition in mice [20, 40]. In this model we have previously shown that HFD feeding impairs hippocampal dependent memory tasks in the form of novel object recognition testing and Morris water maze [20]. To examine potential compounding effects of age, here we assessed cognitive impairment by training mice to associate a context and tone with a foot-shock. All mice were capable of learning and remembering this relationship. However, HFD mice, particularly aged HFD mice, exhibited higher levels of freezing when returned to the training context but in the absence of the tone. As the levels of freezing in response to the tone were similar between groups, this enhanced freezing is likely not due to a generalized increase in fear. While aged HFD mice exhibited extinction learning, their freezing levels remained elevated compared to all other groups. This deficit appears to be specific to extinction learning and are consistent with others suggesting that inflammation within the hippocampus [48] and increased cytokine levels [49] produce similar impairments in fear extinction.

Few studies have assessed cognitive effects of HFD in an age dependent manner. In rats [50], similar to the results presented here, age exacerbated negative cognitive outcomes in animals fed HFD for only 3 days. However, these aged HFD rats froze less compared to their SD counterparts [50], whereas HFD increased freezing in our study. This discrepancy may be due to differences in diet duration or model system (*i.e.*, rats vs mice). In mice, there are conflicting results regarding HFD effects and age on cognitive function [23, 51]. One study reported age worsened HFD hippocampal dependent learning deficits as assessed by elevated plus maze [51]. However, another study showed increased anxiety only in adult HFD mice versus adult controls, and spatial cognitive deficits in aged mice, which diet did not affect [23]. These conflicting results may be due to multiple factors, *e.g*., different age of diet initiation, diet duration, cognitive testing modalities. Normal age-related cognitive changes could also mask subtle HFD effects in older mice. Additionally, behavioral testing is susceptible to variability from multiple factors, such as season, lighting, and light–dark cycles [52, 53].

To investigate underlying neuroinflammatory changes that may contribute to HFD age-dependent fear conditioning deficits, we measured hippocampal cytokine concentrations and inflammatory gene expression. While some studies report increased hippocampal inflammatory cytokines in response to age or HFD [12, 14, 54], we found no changes, which aligns with other published reports [55, 56]. Failure to detect differences in hippocampal cytokines may be due to the high degree of inflammatory regulation required for maintaining homeostasis or the inherent individual variation in inflammatory measures. Others have observed that providing an immune challenge in the form of lipopolysaccharide injection causes a robust increase in inflammatory cytokine production that is impacted by age and HFD [57]. Thus, although we detected no differences in baseline hippocampal cytokines by diet or age, an immune challenge might reveal differences in response to challenge. We did however observe a significant increase in the number of microglia in the hippocampal hilus region in adult mice fed HFD compared to adult SD animals. In addition, a positive correlation was observed between the body weight and microglial numbers, indicating that increased obesity is associated with a greater number of hippocampal microglia. These findings align with our recent report where we show changes in hippocampal microglial activation as measured by microglial morphology after only 4 days using this model of HFD induced obesity and prediabetes [58]. Other have similarly shown that HFD causes robust changes in microglial activation as measured by changes in morphology [12, 22, 55, 59, 60], phagocytosis or phagocytic markers [12, 22, 60], and microglial numbers or density [12, 60, 61]. This in turn contributes to a neuroinflammatory milieu, where increased microglial numbers or activation are associated with increased gene expression of proinflammatory cytokines in the brain [62].

As discussed above, many classic hallmarks of aging, including inflammaging and cellular senescence [63–66], are also associated with obesity and metabolic dysfunction [19, 33]. Indeed, we saw HFD induced a premature aging phenotype by hippocampal RNA-seq. Similarly, hippocampal inflammatory gene expression was age and diet dependent. Specifically, HFD upregulated inflammatory gene expression in adult animals. Age further increased expression, but HFD had the opposite effect in aged mice, decreasing inflammatory gene expression of many of the same DEGs. Broadly, DEGs fell into categories related to lymphocyte differentiation or function, chemotaxis or inflammation, and innate immune cell activation or pattern recognition. Furthermore, several DEGs of interest with this differential regulation were identified; C-X-C motif chemokine 11 (CxCl11), Zinc finger E-box-binding homeobox 1 (Zeb1), and interferon regulatory factor-4 (Irf4).

CxCl11 is a chemokine involved in lymphocyte differentiation or function, which attracts activated T-cells [67]. CxCl11 brain levels increase in response to trauma [68] and in neurological diseases, such as multiple sclerosis [69] and neuroborreliosis [70]. Inflammaging and immunosenescence [71] impact lymphocyte differentiation or function, especially T-cell function, and CxCl11 levels rise in parallel with senescent T-cells in hypertensive patients [72]. The second gene of interest, *Zeb1*, is involved in chemotaxis or inflammation [73], both cornerstones [16–19, 74] of inflammaging and immunosenescence. Zeb1 may regulate adipocyte differentiation in obesity [75, 76] and may also play a role in insulin resistance in adipose tissue [75] and apoptosis in pancreatic beta cell during diabetes [77]. Furthermore, Zeb1 regulates IL-2, which activates innate immune natural killer cells, whose numbers and function decrease with age [78].

The innate immune system is the body’s first line of defense against injury, insult, or infection, and participates in inflammaging and immunosenescence upon continued activation [71]. Irf4, a key player in innate immune responses [79], was the final identified gene of interest. Irf4 helps regulate PGC-1α, a metabolic co-factor that promotes fatty acid oxidation, mitochondrial biogenesis, and brown fat differentiation [80]. Irf4 is expressed by multiple brain cell types, including neurons and microglia, and plays a protective role in response to stroke [81, 82]. Furthermore, and similar to the results here, ischemia in aged mice was associated with a lower IRF4 expression compared to younger animals [83], indicating an age-dependent Irf4 inflammatory response to insult or injury.

Our study has limitations. First, fear conditioning experiments may be limited by known age-associated hearing loss in C57BL/6 mice, which may affect the ability of aged mice to perform the task. However, the 28 kHz, 85 dB tone we used can likely be sensed via vibration. This, combined with similar performances between SD adult and SD aged animals indicates hearing loss likely did not prevent aged mice from forming associative fear memory. Secondly, this study used only male animals. Sex is an important variable in metabolic, immune, and cognitive studies [56, 84, 85]. Given the differences observed here in male mice, future studies are vital to understand the impact of sex on mechanisms leading to cognitive impairment in obesity and metabolic dysfunction.

## Conclusions

Overall, our data demonstrate that age significantly impacts the effect of HFD on the hippocampal inflammatory response and cognitive phenotype, with older aged associated with worse outcomes. Metabolic dysfunction due to HFD is also impacted by age but to a lesser extent, with a potential ‘ceiling effect’ for some metabolic parameters. Hippocampal gene expression supports an age-dependent regulation, which indicate that HFD promotes an early aging phenotype.

## Methods

### Animals and experimental design

Experiments were performed on three cohorts of mice. Cohort 1 comprised young C57BL/6 J males, 5 wk of age (strain # 000,664; Jackson Laboratory, Bar Harbor, ME), whose peripheral metabolic data and neurological tissues were analyzed in a previously published study [86]. Cohort 1 animals were fed ad libitum 10% fat standard diet or 60% high-fat diet (standard diet, SD, D12450B; high-fat diet, HFD, D12492; Research diets, New Brunswick, NJ), and used in this study only to obtain hippocampal tissue for RNA-seq analysis. Cohort 2 included C57BL/6 J males at both 5 wk of age (strain # 000,664; Jackson Laboratory, Bar Harbor, ME) and 1 year (yr) of age (National Institute of Aging aged rodent colony). Cohort 2 animals were used for metabolic phenotyping (body weight, body composition, glucose tolerance testing, plasma insulin, liver pathology, and adipocyte hypertrophy), cognitive phenotyping (fear conditioning), Nanostring hippocampal inflammatory gene expression, and immunologic phenotyping (plasma and hippocampal cytokines). Cohort 2 young and aged mice were fed ad libitum either 10% fat SD or 60% HFD (SD, D12450J; HFD, D12492; Research diets, New Brunswick, NJ). Cohort 3 included C57BL/6 J males either 5 wk (strain # 000,664; Jackson Laboratory, Bar Harbor, ME) or 1 yr of age (National Institute of Aging aged rodent colony). As with cohort 2, cohort 3 young and aged mice were fed ad libitum either 10% fat SD or 60% HFD (SD, D12450J; HFD, D12492; Research diets, New Brunswick, NJ). All animals were acclimated at the University of Michigan for at least 1 wk prior to dietary changes. Mice were also provided water ad libitum in a pathogen-free room maintained under a 14:10 light:dark cycle at 20 ± 2 °C and monitored daily by veterinary staff at the University of Michigan’s Unit for Laboratory Animal Medicine.

The three cohorts differed in diet duration (SD or HFD) and experiment. Cohort 1 young mice were fed SD or HFD for 11 wk or 19 wk for two terminal timepoints to perform hippocampal RNA-seq. Cohort 2 young and aged mice were fed SD or HFD for 14 wk for a single terminal timepoint. Cohort 3 young and aged mice were fed SD or HFD for 16 wk for a single terminal timepoint. At terminal timepoints in all cohorts, animals were sacrificed using an intraperitoneal injection of 150 mg/kg sodium pentobarbital (Fatal-Plus, Vortech Pharmaceuticals, Dearborn, MI). At sacrifice, cohort 1 mice were 16 wk of age after 11 wk on diet, or 24 wk of age after 19 wk on diet; cohort 2 mice were 19 wk of age after 14 wk on diet, or 66 wk of age after 14 wk on diet. Following sacrifice for all animals, plasma was taken for metabolic phenotyping and mice were then perfused with phosphate-buffered saline and tissues harvested. Cohort 1 hippocampal tissue was isolated and snap frozen and stored at -80° C for later RNA extraction and RNA-seq. Cohort 2 terminal plasma was isolated to measure insulin and cytokine levels, hippocampal tissue was isolated, snap frozen, and stored for later cytokine measures or RNA extraction for NanoString inflammatory gene expression analysis, and liver and epididymal fat tissues were formalin fixed for histological analysis. Cohort 3 brains were fixed in 4% paraformaldehyde for later immunohistochemistry. All procedures were carried out per the University of Michigan’s Committee on Use and Care of Animals under protocol numbers PRO0010039, PRO00010247, PRO00006140, and PRO00008116.

### Metabolic phenotyping

Cohort 1 mice underwent metabolic phenotyping as previously reported [86]. Cohort 2 young and aged mice underwent terminal metabolic phenotyping after 14 wk on diet. Metabolic phenotyping was performed on all animals according to the Diabetic Complications Consortium guidelines (https://www.diacomp.org/share/protocols.aspx) and as previously published [30, 31]. At terminal, animals were weighed and glucose tolerance tests were performed, as previously published [30]. Briefly, mice underwent an intraperitoneal injection of 1 g glucose per 1 kg body weight and blood glucose readings were recorded prior to injection and at 15, 30, 60, and 120 min (min) post injection.

Additional metabolic phenotyping for cohort 2 included body composition quantification, plasma insulin concentration, liver pathology scoring, and adipose tissue histomorphometry. Body composition analysis was performed after cognitive phenotyping and immediately prior to study termination at 14 wk using a EcoMRI 4in1-900 (EcchoMRI LLC, Houston, TX) at the Metabolism, Bariatric Surgery and Behavior core as part of the University of Michigan Mouse Metabolic Phenotyping core. Terminal plasma insulin concentrations were measured using a rat/mouse insulin ELISA (catalog # EZRMI-13 k, Millipore Sigma-Aldrich, St. Louis, MO) by the University of Michigan Mouse Metabolic Phenotyping core. Formalin fixed liver tissue samples collected at study endpoint after 14 wk of diet were processed by the University of Michigan in vivo animal core and assessed for liver pathology, which included measures of macrosteatosis (droplet counts within 100–1000 μm^2^ in area, normalized to tissue area) and Kleiner scoring (32–34). Kleiner scoring included measures of lobular inflammation (scale of 0–3), ballooning degeneration (scale of 0–2), steatosis (scale of 0–3), and a summed non-alcoholic fatty liver disease (NAFLD) activity score (NAS) (scale of 0–8).

Formalin fixed epididymal white adipose tissues collected at study endpoint after 14 wk of diet were paraffin embedded, sectioned, stained with hematoxylin and eosin, and assessed for fat histomorphometry, as previously published [87, 88]. Briefly, four representative images were taken per animal at a 10X magnification, and histomorphological analysis was performed using Metamorph software version 7.10.3.279. Images were thresholded to include adipocytes with a shape factor between 0.35 and 1 (shape factor of 0 being a straight line, shape factor of 1 being a perfect circle), an equivalent sphere surface area between 5,000 μm^2^ and 1 × 10^6^ μm^2^, and areas between 10 μm^2^ and 1.5 × 10^3^ μm^2^. Objects bordering the edge of the image were excluded. Following initial thresholding, manual adjustments were made to add, remove, cut, or join adipocytes. For each image, adipocytes were binned from 0 to 2 × 10^4^ μm^2^ at 250 μm^2^ increments. Using the frequency for each binned adipocyte size, the percentage of adipocytes belonging to each bin was calculated for each image and the images for each experimental group were averaged to determine differences for each binned adipocyte size between groups.

## Cognitive phenotyping

Cohort 2 young and aged mice on SD and HFD underwent fear conditioning prior to study termination after 14 wk on diet. Fear conditioning was carried out as previously published [89, 90]. In brief, mice were trained to anticipate a foot shock by training with a 180 s baseline, a tone (28 kHz, 85 dB, 30 s), followed by the 0.75 mA foot shock. Tone/shock pairings were completed 2 additional times, for a total of 3 pairings with a 120 s gap following each tone. On days 2, 3, and 4, mice were placed into the chamber for a total of 30 min, with no tones or shocks to assess fear extinction. The first 5 min of time in the chamber on day 2 was used to assess contextual memory. On day 5, animals were assessed for cued (tone) memory. The chamber was re-configured to represent a different context, *i.e*., different flooring type and wall shape. In addition, the background odor, noise, and lighting were altered [89, 90]. The mice were placed into the reconfigured chamber and given a 180 s baseline, followed by a 28 kHz tone (30 s). Freezing behavior, *i.e*., the absence of movement, excluding breathing, was measured and used to calculate percent freezing, *i.e.*, the amount of time spent freezing out of the total amount of time while in the chamber. Percent freezing was used to assess fear extinction, cued memory, and contextual memory.

### Gene expression

Hippocampi from mice on SD and HFD from cohorts 1 and 2 underwent RNA extraction and gene expression analysis. Cohort 1 hippocampal tissues were collected at study endpoint after 11 wk or 19 wk of diet and processed for RNA-seq, as previously published [31]. Cohort 2 hippocampi were collected at study endpoint after 14 wk of diet and RNA extracted for NanoString nCounter transcriptomics analysis (NanoString Technologies, Seattle, WA). For both cohorts, RNA was isolated using an RNAeasy kit (Qiagen, Germantown, MD), per the manufacturer’s instructions.

Briefly, for RNA-seq on cohort 1 hippocampi, RNA quality was assessed using a 2100 Bioanalyzer at the University of Michigan’s Advanced Genomics Core and used to construct a library, which was sequenced using the NovaSeq 6000 (Illumina, San Diego, CA) to obtain approximately 60 million 50 bp paired-end reads per sample. The raw FASTQ files were first cleaned by removing low quality reads (Q < 30) and adapters with Trimmomatic [91]. All clean reads were mapped to the mouse reference genome mm10 (GRCm38) using HISAT2 mapper [92]. FeatureCounts [93] was used to summarize the reads mapped to mouse genes. Fragments per kilobase of transcript per million mapped reads values were calculated for all genes to represent their expression levels.

For NanoString on cohort 2 hippocampi, RNA samples were sent to Michigan State University for nCounter analysis using NanoString’s mouse immunology panel (catalog # PLS PPL M IMM) with 19 spike-in genes (Iba1, Aim2, Atf6, Cd200r, cGAS, Decaf1, Chop, Dgat2, Perk, Elovl6, Ire1a, Gfap, Jnk, Mapt, Mmp12, Nlrp3, Asc, Scd1, Tmem119). NanoString data were processed using nSolver 4.0 software. Any samples not passing quality control were removed and background thresholding was performed so that any samples with counts below the lowest negative control (a relative gene expression level of 4 counts) were set to that value for analysis. Data were then normalized to the positive controls and to the housekeeping genes provided within the panel. Normalized data were then used for subsequent statistical analysis of relative gene expression (counts).

### Immunological phenotyping

Terminal plasma and hippocampal lysates from cohort 2 young and aged mice on SD and HFD were used to measure cytokine concentrations via enzyme-linked immunosorbent assay (ELISA). Plasma was analyzed for tumor necrosis factor alpha (TNF-α), monocyte chemoattractant protein-1 (MCP-1), interleukin 6 (IL-6), and interleukin 1 beta (IL-1β). Hippocampal lysates were analyzed for TNF-α, MCP-1, IL-6, IL-1β, interferon gamma (IFN-γ), and interleukin 10 (IL-10). ELISA was performed by the University of Michigan Immune Monitoring Core of the Rogel Cancer Center. Terminal IHC for analysis of hippocampal microglial infiltration was performed on cohort 3 hemi brains similar to previous [58]. In brief, following sacrifice, hemi-brains were dissected and fixed in 4% paraformaldehyde for a minimum of 48 h, then passed through a sucrose gradient. Brains were then embedded in OCT and frozen at -80 °C for later sectioning and staining with IBA-1 (rabbit anti-Iba1, 1:1000; catalog # 019–19,741, Wako, Richmond, VA) for microglia and Hoechst for nuclear staining. Images were taken on a Leica Stellaris 8 Falcon confocal microscope with a 40X objective used to take 30 µm Z-stack images. Images were used to count the number of microglia in the medial area of the hilus nearest to the CA4 region of the hippocampus.

### Bioinformatics and statistical analysis

Differentially expressed gene analysis for RNA-seq data was performed with DESeq2 package [94]. Differentially expressed genes (DEGs) were identified with an adjusted *P*-value < 0.05. To identify the overrepresented biological functions, the Kyoto Encyclopedia of Genes and Genomes (KEGG) pathways and Gene Ontology (GO) enrichment analysis were performed using a hypergeometric test with our in-house R analysis package richR (http://github.com/hurlab/richR). The terms with Benjamini–Hochberg corrected *P*-values < 0.05 were deemed as significantly overrepresented biological functions in each DEG set.

Statistical analysis of all other data was performed using either Prism (version 9; GraphPad Software, La Jolla, CA, USA) or SAS 9.4 software (SAS Institute, Cary, NC). GraphPad analyses were performed using either t-test or analysis of variance (ANOVA) and significance of multiple comparisons determined using Tukey’s test. SAS analyses were performed using the Proc Mixed function, and for NanoString data cartridge was set as a random effect to account for potential differences between batches. Normality was established using Anderson–Darling, D'Agostino-Pearson omnibus, Shapiro–Wilk, and Kolmogorov–Smirnov tests. Non-normal data were log transformed and if log transformation did not result in normality, non-parametric analysis was performed using Kruskal–Wallis test. Data are presented as either means or least square means ± standard deviation or as mean ± SEM and are indicated as such in the figure legends.

### Supplementary Information

**Additional file 1. **

**Additional file 2.** 

**Additional file 3.**

**Additional file 4.** 

**Additional file 5.**

## Data Availability

The dataset(s) supporting the conclusions of this article is(are) included within the article (and its additional file(s)).

## References

[1] Blüher M (2019). Obesity: global epidemiology and pathogenesis. Nature Reviews Endocrinology..

[2] Larsson U, Karlsson J, Sullivan M (2002). PAPER Impact of overweight and obesity on health-related quality of life-a Swedish population study. Int J Obes.

[3] Friedlander SL (2003). Decreased Quality of Life Associated With Obesity in School-aged Children. Arch Pediatr Adolesc Med.

[4] Stephenson J (2021). The association between obesity and quality of life: a retrospective analysis of a large-scale population-based cohort study. BMC Public Health.

[5] Pedditizi E, Peters R, Beckett N (2016). The risk of overweight/obesity in mid-life and late life for the development of dementia: a systematic review and meta-analysis of longitudinal studies. Age Ageing.

[6] Callaghan BC (2020). The Prevalence and Determinants of Cognitive Deficits and Traditional Diabetic Complications in the Severely Obese. Diabetes Care.

[7] Hruby A (2016). Determinants and consequences of obesity. Am J Public Health.

[8] Xu WL, Atti AR, Gatz M, Pedersen NL, Johansson B, Fratiglioni L. Midlife overweight and obesity increase late-life dementia risk: a population-based twin study. Neurology. 2011;76(18):1568–74. 10.1212/WNL.0b013e3182190d09 PMC3100125 21536637

[9] de Heredia FP, Gómez-Martínez S, Marcos A. Obesity, inflammation and the immune system. Proc Nutr Soc. 2012;71(2):332–8. 10.1017/S0029665112000092 22429824

[10] Van Eldik LJ (2016). The roles of inflammation and immune mechanisms in Alzheimer's disease. Alzheimer's & Dementia : Translational Research & Clinical Interventions.

[11] Cope EC (2018). Microglia Play an Active Role in Obesity-Associated Cognitive Decline. J Neurosci.

[12] Hao S (2016). Dietary obesity reversibly induces synaptic stripping by microglia and impairs hippocampal plasticity. Brain Behav Immun.

[13] Rohm TV (2022). Inflammation in obesity, diabetes, and related disorders. Immunity.

[14] Nakandakari SCBR (2019). Short-term high-fat diet modulates several inflammatory, ER stress, and apoptosis markers in the hippocampus of young mice. Brain Behav Immun.

[15] Halliday G (2017). Pathology and hippocampal atrophy in Alzheimer's disease. The Lancet Neurology.

[16] Weyand CM, Goronzy JJ (2016). Aging of the immune system: Mechanisms and therapeutic targets. Ann Am Thorac Soc.

[17] Haynes L (2020). Aging of the Immune System: Research Challenges to Enhance the Health Span of Older Adults. Front Aging..

[18] Nikolich-Žugich J (2017). The twilight of immunity: emerging concepts in aging of the immune system. Nat Immunol..

[19] Franceschi C (2018). Inflammaging: a new immune-metabolic viewpoint for age-related diseases. Nat Rev Endocrinol.

[20] Sims-Robinson C (2016). Dietary reversal ameliorates short-and long-term memory deficits induced by high-fat diet early in life. PLoS ONE.

[21] Watson LS (2020). High-Fat diet impairs tactile discrimination memory in the mouse. Behav Brain Res..

[22] Zhuang H (2022). Long-term high-fat diet consumption by mice throughout adulthood induces neurobehavioral alterations and hippocampal neuronal remodeling accompanied by augmented microglial lipid accumulation. Brain Behav Immun.

[23] Kesby JP (2015). Spatial Cognition in Adult and Aged Mice Exposed to High-Fat Diet. PLoS ONE.

[24] Tucsek Z (2014). Obesity in aging exacerbates blood–brain barrier disruption, neuroinflammation, and oxidative stress in the mouse hippocampus: effects on expression of genes involved in beta-amyloid generation and Alzheimer’s disease. Journals of Gerontology Series A: Biomedical Sciences and Medical Sciences.

[25] Julien C (2010). High-fat diet aggravates amyloid-beta and tau pathologies in the 3xTg-AD mouse model. Neurobiol Aging.

[26] Gannon OJ (2022). High-fat diet exacerbates cognitive decline in mouse models of Alzheimer's disease and mixed dementia in a sex-dependent manner. J Neuroinflammation..

[27] Moser VA, Pike CJ (2017). Obesity Accelerates Alzheimer-Related Pathology in APOE4 but not APOE3 Mice. eNeuro.

[28] Spencer SJ (2019). High-fat diet worsens the impact of aging on microglial function and morphology in a region-specific manner. Neurobiol Aging.

[29] Valcarcel-Ares MN (2019). Obesity in Aging Exacerbates Neuroinflammation, Dysregulating Synaptic Function-Related Genes and Altering Eicosanoid Synthesis in the Mouse Hippocampus: Potential Role in Impaired Synaptic Plasticity and Cognitive Decline. The Journals of Gerontology: Series A.

[30] O'Brien PD (2018). Juvenile murine models of prediabetes and type 2 diabetes develop neuropathy. Dis Model Mech..

[31] O'Brien PD (2020). Integrated lipidomic and transcriptomic analyses identify altered nerve triglycerides in mouse models of prediabetes and type 2 diabetes. Dis Model Mech.

[32] Ximerakis M (2019). Single-cell transcriptomic profiling of the aging mouse brain. Nat Neurosci.

[33] Ahima RS. Connecting obesity, aging and diabetes. Nat Med. 2009;15(9):996–7. 10.1038/nm0909-996 19734871

[34] Chen G, Yung R (2019). Meta-inflammaging at the crossroad of geroscience. Aging med (Milton (N.S.W))..

[35] Hotamisligil GS (2017). Inflammation, metaflammation and immunometabolic disorders. Nature..

[36] Fessler MB, Rudel LL, Brown M (2009). Toll-like receptor signaling links dietary fatty acids to the metabolic syndrome. Curr Opin Lipidol.

[37] Li B (2020). A global perspective on the crosstalk between saturated fatty acids and Toll-like receptor 4 in the etiology of inflammation and insulin resistance. Prog lipid res..

[38] Arkan MC, Hevener AL, Greten FR, Maeda S, Li ZW, Long JM, Karin M. IKK-β links inflammation to obesity-induced insulin resistance. Nat Med. 2005;11(2):191–8. 10.1038/nm1185 15685170

[39] Vanani AR (2021). Dimethyl fumarate reduces oxidative stress, inflammation and fat deposition by modulation of Nrf2, SREBP-1c and NF-κB signaling in HFD fed mice. Life Sci..

[40] Jeon BT (2012). Resveratrol Attenuates Obesity-Associated Peripheral and Central Inflammation and Improves Memory Deficit in Mice Fed a High-Fat Diet. Diabetes.

[41] Henn RE (2022). Glial-neuron crosstalk in health and disease: A focus on metabolism, obesity, and cognitive impairment. Neurobiol Dis..

[42] Biessels GJ, Bravenboer B, Gispen WH (2004). Glucose, insulin and the brain: modulation of cognition and synaptic plasticity in health and disease: a preface. Eur J Pharmacol..

[43] Ahmad RMAH, Nida'a AA Nida'a, Domi HA Al (2022). Brain insulin resistance as a mechanistic mediator links peripheral metabolic disorders with declining cognition. Diabetes Metab Synd..

[44] Képes Z (2021). Glucose-level dependent brain hypometabolism in type 2 diabetes mellitus and obesity. European Journal of Hybrid Imaging.

[45] Henn RE (2022). Glial-neuron crosstalk in health and disease: A focus on metabolism, obesity, and cognitive impairment. Neurobiol Dis.

[46] Więckowska-Gacek A (2021). Western diet as a trigger of Alzheimer’s disease: From metabolic syndrome and systemic inflammation to neuroinflammation and neurodegeneration. Ageing Res Rev.

[47] Sheikh MH (2022). Impact of metabolic disorders on the structural, functional, and immunological integrity of the blood-brain barrier: Therapeutic avenues. FASEB J.

[48] Dong Y (2020). Stress-induced NLRP3 inflammasome activation negatively regulates fear memory in mice. J Neuroinflammation.

[49] Singer BH (2016). Cecal Ligation and Puncture Results in Long-Term Central Nervous System Myeloid Inflammation. PLoS ONE.

[50] Spencer SJ (2017). High-fat diet and aging interact to produce neuroinflammation and impair hippocampal-and amygdalar-dependent memory. Neurobiol Aging.

[51] Tucsek Z (2014). Aging exacerbates obesity-induced cerebromicrovascular rarefaction, neurovascular uncoupling, and cognitive decline in mice. J Gerontol A Biol Sci Med Sci..

[52] Schellinck HM, Cyr DP, Brown RE (2010). How Many Ways Can Mouse Behavioral Experiments Go Wrong? Confounding Variables in Mouse Models of Neurodegenerative Diseases and How to Control Them. Advances in the Study of Behavior.

[53] Roedel A (2006). Effects of light or dark phase testing on behavioural and cognitive performance in DBA mice. Lab Anim.

[54] Kang EB, Koo JH, Jang YC, Yang CH, Lee Y, Cosio‐Lima L.M, Cho JY. Neuroprotective Effects of Endurance Exercise Against High-Fat Diet-Induced Hippocampal Neuroinflammation. J Neuroendocrinol. 2016;28(5). 10.1111/jne.12385 26991447

[55] Daly CM (2022). Sex differences in response to a high fat, high sucrose diet in both the gut microbiome and hypothalamic astrocytes and microglia. Nutr Neurosci.

[56] Church JS, Renzelman ML, Schwartzer JJ (2022). Ten-week high fat and high sugar diets in mice alter gut-brain axis cytokines in a sex-dependent manner. J Nutr Biochem.

[57] Boitard C (2014). Impairment of hippocampal-dependent memory induced by juvenile high-fat diet intake is associated with enhanced hippocampal inflammation in rats. Brain Behav Immun.

[58] Elzinga SE (2022). cGAS/STING and innate brain inflammation following acute high-fat feeding. Front Immunol..

[59] Gzielo K (2017). Long-term consumption of high-fat diet in rats: effects on microglial and astrocytic morphology and neuronal nitric oxide synthase expression. Cell Mol Neurobiol.

[60] Yao X (2022). High-Fat Diet Consumption in Adolescence Induces Emotional Behavior Alterations and Hippocampal Neurogenesis Deficits Accompanied by Excessive Microglial Activation. Int J Mol Sci.

[61] Gao Y (2014). Hormones and diet, but not body weight, control hypothalamic microglial activity. Glia.

[62] Mendes NF (2018). Hypothalamic microglial activation in obesity: A mini-review. Front Neurosci.

[63] Chalan P (2015). Rheumatoid Arthritis, Immunosenescence and the Hallmarks of Aging. Curr Aging Sci.

[64] De Martinis M (2005). Inflamm-ageing and lifelong antigenic load as major determinants of ageing rate and longevity. FEBS Lett.

[65] Freund A (2010). Inflammatory networks during cellular senescence: causes and consequences. Trends Mol Med.

[66] Tchkonia T (2013). Cellular senescence and the senescent secretory phenotype: therapeutic opportunities. J Clin Investig.

[67] Cole KE (1998). Interferon-inducible T Cell Alpha Chemoattractant (I-TAC): A Novel Non-ELR CXC Chemokine with Potent Activity on Activated T Cells through Selective High Affinity Binding to CXCR3. J Exp Med..

[68] Mousessian AS (2021). CXCR7, CXCR4, and Their Ligand Expression Profile in Traumatic Brain Injury. World Neurosurgery.

[69] Mccoll SR (2004). Expression of rat I-TAC/CXCL11/SCYA11 during central nervous system inflammation: comparison with other CXCR3 ligands. Lab Invest.

[70] Rupprecht TA (2005). ORIGINAL COMMUNICATION. J Neurol.

[71] Thomas R, Wang W, Su DM (2020). Contributions of Age-Related Thymic Involution to Immunosenescence and Inflammaging. Immun Ageing.

[72] Yu HT (2016). T cell senescence and cardiovascular diseases. Clin Exp Med.

[73] Wang J (2009). The transcription repressor, ZEB1, cooperates with CtBP2 and HDAC1 to suppress IL-2 gene activation in T cells. Int Immunol.

[74] Bleve A (2016). Immunosenescence. Inflammaging, and Frailty: Role of Myeloid Cells in Age-Related Diseases.

[75] Becker-Greene D (2021). MiR-409–3p targets a MAP4K3-ZEB1-PLGF signaling axis and controls brown adipose tissue angiogenesis and insulin resistance. Cell Mol Life Sci..

[76] Gubelmann C (2014). Identification of the transcription factor ZEB1 as a central component of the adipogenic gene regulatory network. eLife.

[77] Filios SR (2014). MicroRNA-200 is induced by thioredoxin-interacting protein and regulates Zeb1 protein signaling and beta cell. J Biol Chem.

[78] Panda A (2009). Human innate immunosenescence: causes and consequences for immunity in old age. Trends Immunol.

[79] Klein U, Casola S, Cattoretti G, Shen Q, Lia M, Mo T, Dalla-Favera R. Transcription factor IRF4 controls plasma cell differentiation and class-switch recombination. Nat Immunol. 2006;7(7):773–82. 10.1038/ni1357 16767092

[80] Kong X (2014). IRF4 is a key thermogenic transcriptional partner of PGC-1α. Cell.

[81] Guo S (2014). IRF4 is a novel mediator for neuronal survival in ischaemic stroke. Cell Death Differ.

[82] Al Mamun A (2020). Microglial IRF5-IRF4 regulatory axis regulates neuroinflammation after cerebral ischemia and impacts stroke outcomes. Proc Natl Acad Sci USA.

[83] Zhao SC (2017). Age-related differences in interferon regulatory factor-4 and -5 signaling in ischemic brains of mice. Acta Pharmacol Sin.

[84] Barron AM (2013). Sex-specific effects of high fat diet on indices of metabolic syndrome in 3xTg-AD mice: implications for Alzheimer's disease. PloS one..

[85] Elzinga SE (2021). Sex differences in insulin resistance, but not peripheral neuropathy, in a diet-induced prediabetes mouse model. Dis Model Mech..

[86] O’Brien PD (2020). Integrated lipidomic and transcriptomic analyses identify altered nerve triglycerides in mouse models of prediabetes and type 2 diabetes. Dis Model Mech.

[87] Hinder LM (2017). Dietary reversal of neuropathy in a murine model of prediabetes and metabolic syndrome. Dis Model Mech.

[88] Parlee SD (2014). Quantifying size and number of adipocytes in adipose tissue. Methods in enzymology.

[89] Cazares VA, Rodriguez G, Parent R, Ouillette L, Glanowska KM, Moore SJ, Murphy GG. Environmental variables that ameliorate extinction learning deficits in the 129S1/SvlmJ mouse strain. Genes Brain Behav. 2019;18(7):e12575. 10.1111/gbb.12575 PMC6718342 30973205

[90] Temme SJ, Murphy GG (2017). The L-type voltage-gated calcium channel Ca V 1.2 mediates fear extinction and modulates synaptic tone in the lateral amygdala. Learn Mem..

[91] Bolger AM, Lohse M, Usadel B (2014). Genome analysis Trimmomatic: a flexible trimmer for Illumina sequence data.

[92] Kim D (2019). Graph-based genome alignment and genotyping with HISAT2 and HISAT-genotype. Nat Biotechnol..

[93] Liao Y, Smyth GK, Shi W (2014). Sequence analysis featureCounts: an efficient general purpose program for assigning sequence reads to genomic features.

[94] Love MI, Huber W, Anders S (2014). Moderated estimation of fold change and dispersion for RNA-seq data with DESeq2. Genome Biol.

