# Correction: Obesity-induced neuroinflammation and cognitive impairment in young adult versus middle-aged mice

**DOI:** 10.1186/s12979-024-00422-7

**Published:** 2024-02-21

**Authors:** Rosemary E. Henn, Sarah E. Elzinga, Emily Glass, Rachel Parent, Kai Guo, Adam M. Allouch, Faye E. Mendelson, John Hayes, Ian Webber-Davis, Geoffery G. Murphy, Junguk Hur, Eva L. Feldman

**Affiliations:** 1 Department of Neurology, University of Michigan, Ann Arbor, MI 48109 USA; 2 NeuroNetwork for Emerging Therapies, University of Michigan, Ann Arbor, MI 48109 USA; 3 Michigan Neuroscience Institute, University of Michigan, Ann Arbor, MI 48109 USA; 4 Department of Molecular and Integrative Physiology, Division of Cardiovascular Medicine, University of Michigan, Ann Arbor, MI 48109 USA; 5 Department of Biomedical Sciences, University of North Dakota, Grand Forks, ND 58202 USA

**Correction: Immun Ageing 19, 67 (2022)**

10.1186/s12979-022-00323-7

Following publication of the original article [1], the authors identified an error in the author name of Adam M. Allouch.

The incorrect author name is: Adam A. Allouch.

The correct author name is: Adam M. Allouch.

The author group has been updated above and the original article [1] has been corrected.
