# Supplementary figures and images for: Obesity-induced neuroinflammation and cognitive impairment in young adult versus middle-aged mice
Source: Immun Ageing. 2022 Dec 22;19:67. doi: 10.1186/s12979-022-00323-7 (PMC9773607; doi:10.1186/s12979-022-00323-7)

**
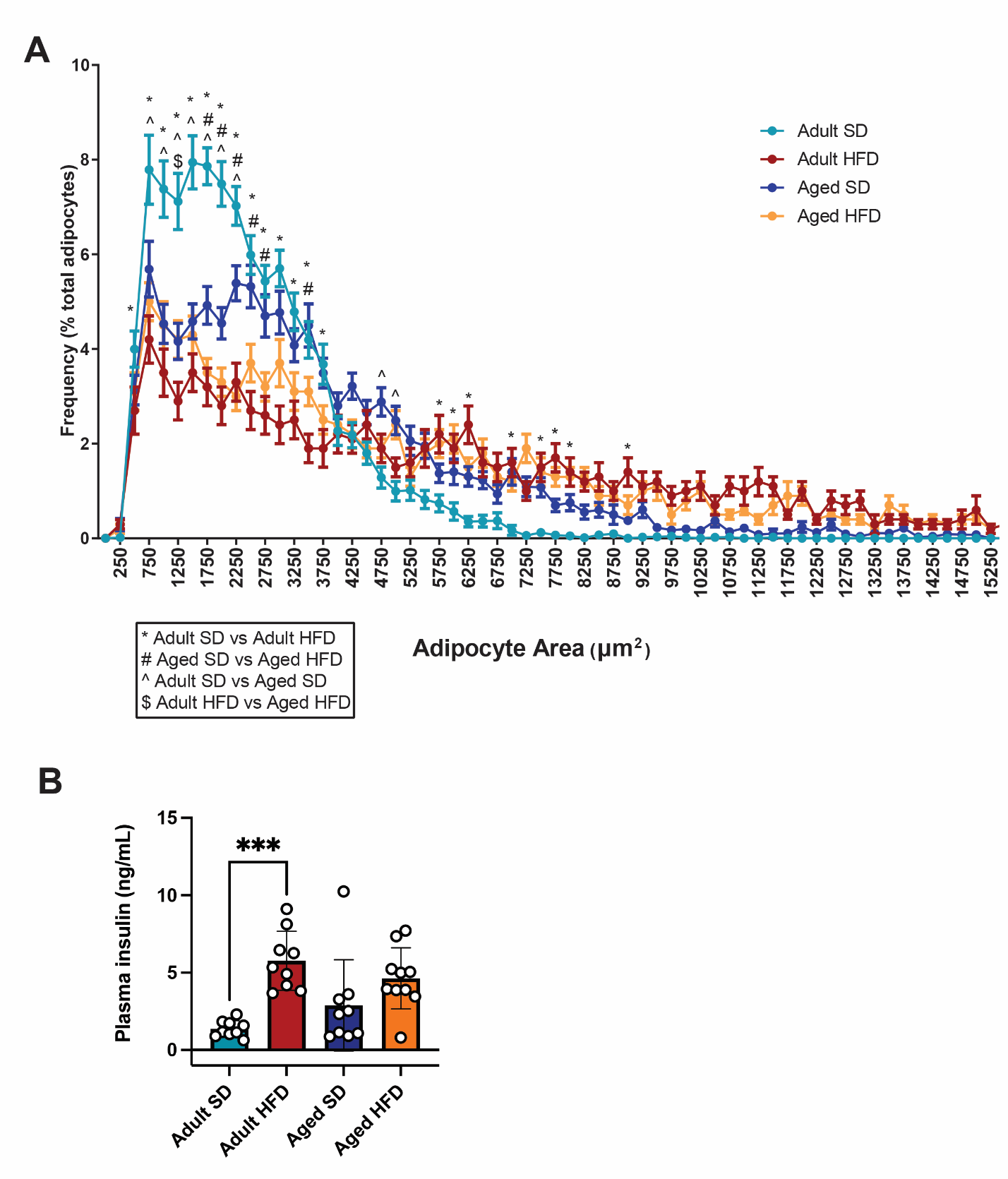
**

**Additional Figure 1. Terminal adipocyte hypertrophy and plasma insulin.**

Supplement: Supplementary file 1 — Additional file 1. [file 12979_2022_323_MOESM1_ESM.docx]

**
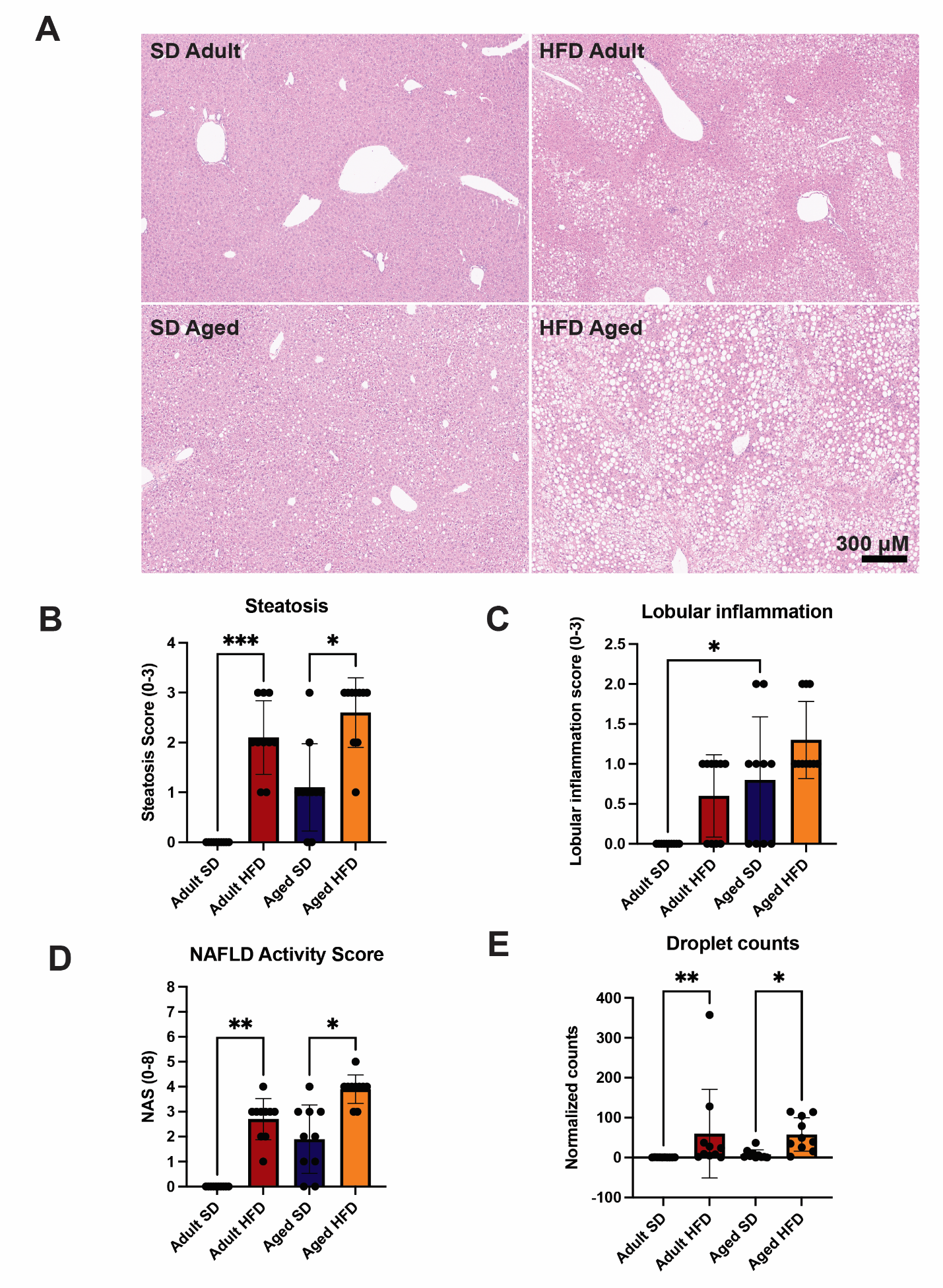
**

**Additional Figure 2. Terminal liver Pathology.**

Supplement: Supplementary file 2 — Additional file 2. [file 12979_2022_323_MOESM2_ESM.docx]

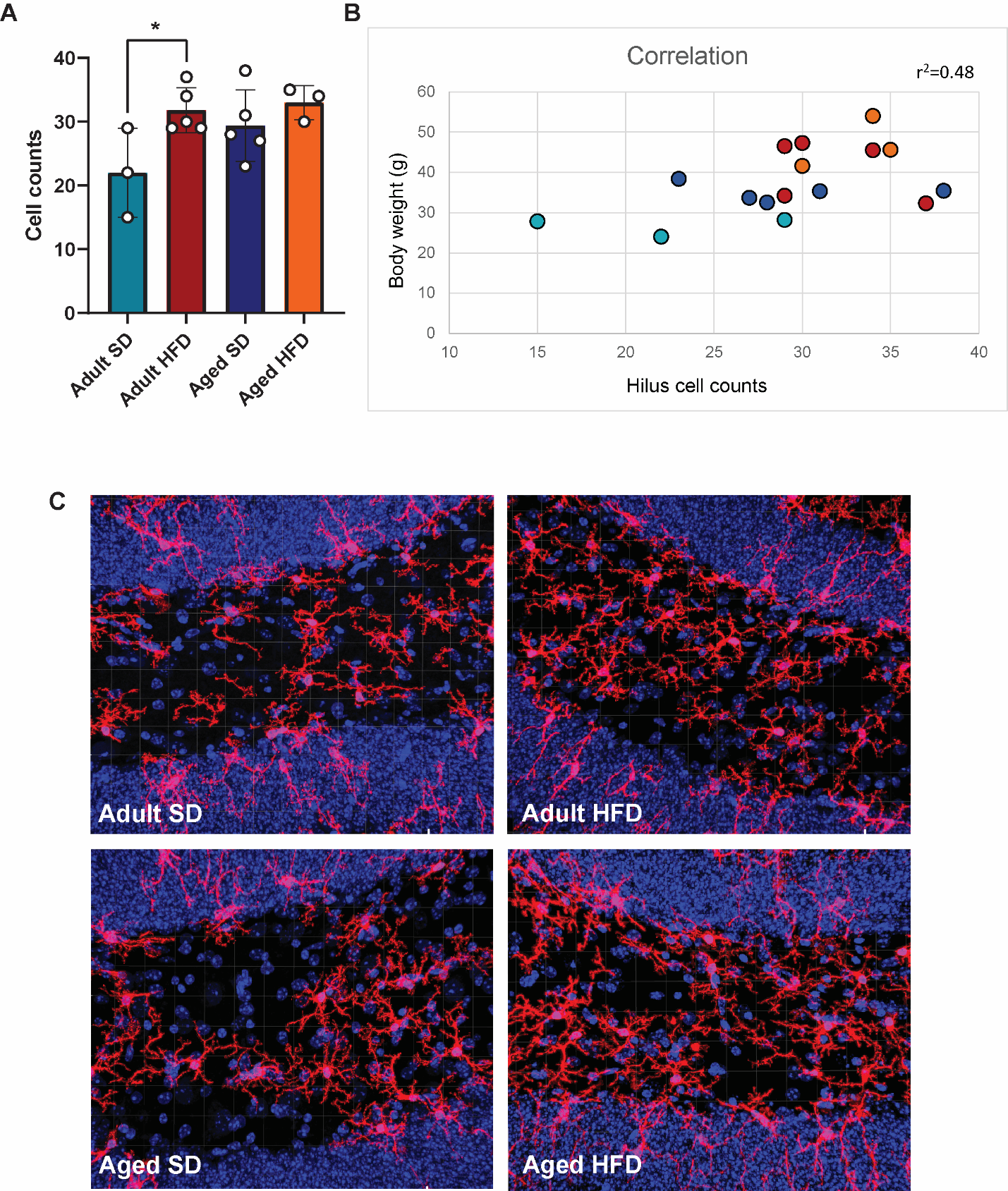


**Additional Figure 3. Hippocampal microglial numbers.**

Supplement: Supplementary file 3 — Additional file 3. [file 12979_2022_323_MOESM3_ESM.docx]
